# Delineating reef fish trophic guilds with global gut content data synthesis and phylogeny

**DOI:** 10.1371/journal.pbio.3000702

**Published:** 2020-12-28

**Authors:** Valeriano Parravicini, Jordan M. Casey, Nina M. D. Schiettekatte, Simon J. Brandl, Chloé Pozas-Schacre, Jérémy Carlot, Graham J. Edgar, Nicholas A. J. Graham, Mireille Harmelin-Vivien, Michel Kulbicki, Giovanni Strona, Rick D. Stuart-Smith

**Affiliations:** 1 PSL Université Paris: EPHE-UPVD-CNRS, USR 3278 CRIOBE, Université de Perpignan, Perpignan, France; 2 Laboratoire d’Excellence “CORAIL,” Perpignan, France; 3 Department of Marine Science, University of Texas at Austin, Marine Science Institute, Port Aransas, Texas, United States of America; 4 Centre for the Synthesis and Analysis of Biodiversity (CESAB), Institut Bouisson Bertrand, Montpellier, France; 5 Institute for Marine and Antarctic Studies, University of Tasmania, Hobart, Tasmania, Australia; 6 Lancaster Environment Centre, Lancaster University, Lancaster, United Kingdom; 7 Aix-Marseille Université, Institut Méditerranéen d’Océanologie, CNRS/INSU, Marseille, France; 8 UMR Entropie, LabEx Corail, IRD, Université de Perpignan, Perpignan, France; 9 University of Helsinki, Department of Bioscience, Helsinki, Finland; Estacion Biologica de Doñana CSIC, SPAIN

## Abstract

Understanding species’ roles in food webs requires an accurate assessment of their trophic niche. However, it is challenging to delineate potential trophic interactions across an ecosystem, and a paucity of empirical information often leads to inconsistent definitions of trophic guilds based on expert opinion, especially when applied to hyperdiverse ecosystems. Using coral reef fishes as a model group, we show that experts disagree on the assignment of broad trophic guilds for more than 20% of species, which hampers comparability across studies. Here, we propose a quantitative, unbiased, and reproducible approach to define trophic guilds and apply recent advances in machine learning to predict probabilities of pairwise trophic interactions with high accuracy. We synthesize data from community-wide gut content analyses of tropical coral reef fishes worldwide, resulting in diet information from 13,961 individuals belonging to 615 reef fish. We then use network analysis to identify 8 trophic guilds and Bayesian phylogenetic modeling to show that trophic guilds can be predicted based on phylogeny and maximum body size. Finally, we use machine learning to test whether pairwise trophic interactions can be predicted with accuracy. Our models achieved a misclassification error of less than 5%, indicating that our approach results in a quantitative and reproducible trophic categorization scheme, as well as high-resolution probabilities of trophic interactions. By applying our framework to the most diverse vertebrate consumer group, we show that it can be applied to other organismal groups to advance reproducibility in trait-based ecology. Our work thus provides a viable approach to account for the complexity of predator–prey interactions in highly diverse ecosystems.

## Abstract 2

The diversity of life on our planet has produced a remarkable variety of biological traits that characterize different species, and such traits are widely considered an alternative to taxonomy to increase our understanding of biodiversity and ecosystem functioning. This study presents an unbiased and fully reproducible framework to delineate trophic guilds in reef fishes.

## Introduction

A fundamental goal in ecology is to understand the mechanisms behind the maintenance of biodiversity and ecosystem functioning [1,2]. Understanding the ecological niches of species and their role in ecosystems is central to this endeavor [3,4]. In fact, the degree of niche overlap among species can be a major determinant of relationships among species richness [5], ecosystem productivity [6–8], and vulnerability [9] since limited functional redundancy can make ecosystems more prone to lose entire energetic pathways [10–12]. With growing threats to flora and fauna worldwide, the need to quantify the impact of biodiversity loss has amplified the use of functional groups, which group species (and life history stages) that share common ecological characteristics and are often defined by coarse, categorical descriptors of species traits [13–16].

Natural systems are inherently complex, with almost innumerable, non-random linkages across an intricate network of ecological interactions [17]. Accounting for such complexity is critical to define energetic pathways and, ultimately, ecosystem functioning [18]. However, our understanding of even basic predator–prey interactions is limited for many ecosystems, and expert opinion does not adequately fill this knowledge gap [19]. To overcome this limitation, scientists have developed methods to infer the probability of ecological interactions based on species’ evolutionary history and ecological traits [20–23]. However, predicting trophic interactions across the entire spectrum of potential predator–prey interactions often remains unresolved in hyperdiverse ecosystems. In these cases, categorical traits are frequently used as proxy of both ecosystem functioning and trophic structure [24].

Delineating the ecological niche with discrete categories has several operational advantages. First, grouping species into categories helps decompose highly complex ecosystems into comprehensible units, while traditional taxonomic analyses may be difficult to interpret. Second, ecological predictions tied to species are restricted to the geographic range of the species, whereas predictions of functional groups can be globally comparable. Third, the use of functional groups enables the quantification of functional metrics (e.g., functional richness and functional redundancy) from a standard community data matrix without complex manipulative experiments [25–27]. The promise of “user-friendly” metrics for functional ecology has encouraged the employment of trait-based data in community ecology; even with a paucity of empirical information, it is often assumed that experts can achieve accurate descriptions of the ecological niche of species [25,28,29].

Coral reefs, one of the most diverse ecosystems on Earth, have inspired a plethora of studies that assess ecosystem functioning. However, only few studies have attempted to categorize fluxes on a continuous gradient across an entire food web [30], and most studies use expert opinion to define simple functional groups. Indeed, recent efforts have compiled trait-based datasets for 2 major components of this ecosystem: corals and fishes [31,32]. For some traits, such as maximum body size in fishes, the compilation process is simple and accurate because unidimensional, quantitative data (e.g., maximum total length) are compiled in publicly accessible databases; however, when it comes to species’ diet or behavior, obtaining consensual data is much more difficult. For example, dietary data are multidimensional (i.e., various prey items can be recorded across individuals), influenced by ontogenetic and spatio-temporal variables (i.e., life history, time, and location can incur dietary shifts), and prone to methodological differences and thus observer bias. Therefore, while some exceptions exist [30,33], our capacity to define coral reef trophic interactions still largely depends on discrete trophic categories defined by expert opinion [27].

Although experts sometimes agree on relevant traits to define trophic categories, there is often an implicit disagreement. Across the coral reef literature, the number and resolution of reef fish trophic guilds substantially differs. Studies commonly define 3 [34] to 8 [35] trophic guilds, with particular ambivalence on the resolution at which to define herbivores and invertivores [36–39]. Among all trait classification schemes for reef fishes, only a few are openly accessible (e.g., [39,40]). Consequently, different research groups tend to employ proprietary classifications, with little possibility to cross-check and compare assigned traits. The classification of species into functional groups has advantages for our understanding of ecological patterns [42,43]. However, the lack of agreement and the limited transparency of trait-based datasets can conjure skepticism and inhibit the emergence of general patterns.

Here, we quantify expert agreement in the definition of coral reef fish trophic guilds and propose a novel, quantitative framework to delineate trophic guilds. Moreover, we test whether machine learning allows us to go beyond the definition of discrete categories, accurately predicting individual trophic interactions in hyperdiverse ecosystems. We compiled all quantitative, community-wide dietary analyses from several locations across the Indo-Pacific and the Caribbean. Then, we used network analysis to quantitatively define modules that correspond to trophic guilds and machine learning to infer pairwise trophic interactions. We then examined phylogenetic niche conservatism between species to predict trophic guilds and probabilities of pairwise trophic interactions for the global pool of coral reef fishes. Our framework is fully reproducible and can be extended and updated as new data become available.

## Materials and methods

### Assessment of expert agreement

We systematically searched Google Scholar, including papers since 2000, using the following keywords: “coral reefs” AND “reef fish” AND (“fish community” OR “fish assemblage”) AND “diet” AND (“functional group” OR “functional trait” OR “functional entity” OR “trophic guild” OR “trophic group”). The search yielded 856 papers, which were individually assessed. We only kept studies performed at the community level that targeted all trophic levels. Most studies were excluded because they only included specific families or groups, or the data were not provided with the publication. When the data were not provided with the publications, we contacted authors with trophic classifications used widely used across the literature. We often found redundant results, with groups publishing several papers using the same classification scheme. In those cases, only the most recent reference was retained. Of the 856 papers, 163 papers were inaccessible (i.e., non-English language and/or data inaccessibility despite contacting the first author). Thus, 182 studies met the criteria of our initial assessment, which ultimately yielded 33 papers with independent trophic classifications (S1 Table).

The classifications were not uniform in terms of the number and nature of trophic guilds. In order to compare trophic guilds across publications, we first standardized the schemes by converting the original trophic categories into 5 broad trophic guilds: “herbivores and detritivores,” “invertivores,” “omnivores,” “planktivores,” and “piscivores.” All classification schemes could be attributed to these categories with the exception of 8 papers that did not include either “omnivores” or “piscivores” as a category. In these cases, the comparison was only made across the 4 comparable guilds.

In order to assess expert agreement, we compared each possible pair of classifications that shared at least 50 species, generated a confusion matrix (also known as an error matrix; [50]), and measured agreement as the proportion of species with matching trophic guild assignments. We then calculated the average agreement between classification pairs for each trophic guild. Simplifying categories into 5 comparable, broad trophic guilds therefore reduced the number of trophic categories and naturally inflated agreement among authors; thus, our estimates of author agreement are conservative.

### Data collection on fish gut contents

To provide a quantitative definition of trophic guilds for reef fishes, we collected gut content data across the literature at the individual or species level for Elasmobranchii (i.e., cartilaginous fishes) and Actinopterygii (i.e., ray-finned fishes). We obtained dietary information from 5 published works: Hiatt and Strasburg (1960) for the Marshall Islands [51], Randall (1967) for Puerto Rico and the Virgin Islands [52], Hobson (1974) for Hawaii [53], Harmelin-Vivien (1979) for Madagascar [54], and Sano and colleagues (1984) for Okinawa [55]. In addition, we provide hitherto unpublished data on the gut contents of 3,015 individuals of 111 species collected in New Caledonia from 1984 to 2000.

All dietary information was based on visual gut content analysis that reported prey ingestion as a volumetric percentage or frequency. The data were standardized and analyzed as proportions. To our knowledge, the compiled dataset represents the first detailed synthesis of community-wide visual gut content analyses to infer the structure of coral reef food webs across ocean basins. A total of 13,961 non-empty fish guts belonging to 615 species were analyzed, and more than 1,200 different prey items were described across the original datasets.

First, fish species and family names were taxonomically verified and corrected with the R package *rfishbase* [56]. Only species with at least 10 non-empty guts were kept for further analysis. The taxonomic classification of each prey item was then obtained, and all non-informative and redundant items were discarded (e.g., unidentified fragments; “crustacea fragments” when co-occurring with an item already identified to a lower taxonomic level such as “shrimp”). Prey identification was highly heterogeneous across the 6 datasets, differing in taxonomic level and the use of common or scientific names (e.g., crabs versus Brachyura). In order to make the 6 datasets comparable, prey items were grouped into 38 ecologically informative prey groups (S2 Table). Items were generally assigned to groups corresponding to their phylum or class. Due to the high diversity and detailed descriptions of crustaceans, they were assigned to the level of order or superorder. Most groups follow official taxonomic classifications except for “detritus,” “inorganic,” and “zooplankton.” In the West Indies dataset [52], items labelled as “Algae & Detritus” were assigned to both of the categories “detritus” and “benthic autotroph,” and the percentage was equally divided in 2. The category “zooplankton” includes all eggs and larvae regardless of taxonomy.

### Definition of trophic guilds with network analysis

Of the 615 species with dietary information, 516 were present in only 1 location, 66 were collected in 2 locations, 25 in 3 locations, 7 in 4 locations, and only 1 across 5 locations. We tested whether there was a strong dietary difference in species present in more than 1 location by creating a quantitative bipartite network [57] where fish species at each location were linked to the 38 prey groups. This network was weighted so that edge weights represent the proportional contribution of each prey group to the diet of a species at a given location.

In order to identify network modules that correspond to reef fish trophic guilds and their ingested prey, we used the maximization of the weighted network modularity based on weighted bipartite networks [58]. Due to the high occurrence of accidental predation in reef fishes, we used weighted networks to define modules so that rare or accidental prey would not drive the definition of trophic guilds.

Since the modularity maximization algorithm has an initial random step, it may converge to different (although similar) suboptimal solutions each time the analysis is performed, which is common across several optimization algorithms, such as simulated annealing [59]. To guarantee reproducibility and reduce the risk of basing our analysis on an outlier, we performed the modularity maximization 500 times and retained the medoid solution, which was identified as the solution with the highest similarity to the other 499 modules. Similarity between classifications was assessed as the variation of information, which is an accepted metric to compare multiple clustering results [60]. Overall, 68% of the species found in more than 1 location belonged to the same module. Therefore, we considered the regional effect to be minor and performed the analysis on the global network, ignoring regional variability and increasing the number of individuals per species.

### Phylogenetic conservatism of trophic guilds

We extracted the phylogenetic position of the 615 species used for the definition of trophic guilds through the Fish Tree of Life [61]. A total of 603 out of 615 species were available in the Fish Tree of Life, but only 535 species had verified phylogenetic information. For the taxa available in the Fish Tree of Life without verified phylogenetic information, we retrieved the pseudo-posterior distribution of 100 synthetic stochastically resolved phylogenies where missing taxa were placed according to taxonomy using the function *fishtree_complete_phylogeny()* in the R package *fishtree* [62].

We quantified the phylogenetic conservatism of trophic guilds by calculating the phylogenetic statistic δ, which uses a Bayesian approach for discrete variables [63]. The δ statistic can be arbitrarily large with a high level of variation, depending on the number of species and trait levels. To evaluate the significance of the δ statistic, we applied a bootstrapping approach where we quantified δ 100 times after randomly shuffling the trait values.

We then fitted a multinomial phylogenetic regression to predict fish trophic guild according to phylogeny and body size with the R package *brms* [64]. We used a multinomial logit link function. As such, the probability of a particular trophic guild is computed as follows:

$$Pr(k|{mu}_{1},{mu}_{2},\ldots ,{mu}_{k})=\frac{{mu}_{k}}{\sum_{1}^{k}exp({mu}_{i})}$$ (1)

with *mu*_*k*_ defined as

$${mu}_{1}=0,{mu}_{k|2:8}=\beta_{0k}+\beta_{1k}log(sizemax)+\gamma_{0phy\times k},$$ (2)

where *β*_0*k*_ is the category-specific fixed-effect intercept, *β*_1*k*_ is the slope for the natural transformed maximum body size for each category *k*, and *γ*_0phy×*k*_ is the matrix of random effect coefficients that account for intercept variation based on relatedness as described by the phylogeny for each diet category *k*. We used uninformative priors and ran the model for 3 chains, each with 6,000 iterations and a warm-up of 1,000 iterations. We visualized the fitted probabilities for each trophic guild with a phylogenetic tree, including the 535 species with verified phylogenetic positions using the R package *ggtree* [63]. Next, we used our model to predict the most likely trophic guild for the global pool of reef fish species. For the extrapolation, we selected all species within reef fish families with more than 1 representative species (but we also included *Zanclus cornutus*, which is the only species in the family Zanclidae), which resulted in 50 families. Further, we only selected species with a maximum length greater than 3 cm, which was the maximum size of the smallest fish in our compiled database. This selection process resulted in a list of 4,554 reef fish species.

Currently, no streamlined method exists to predict traits for new species from a phylogenetic regression model. We circumvented this issue by extracting draws of the phylogenetic effect (*γ*_0phy×*k*_) for each species included in the model. We subsequently predicted the phylogenetic effects for missing species with the help of the function *phyEstimate* from the R package *picante* [65]. This function uses phylogenetic ancestral state estimation to infer trait values for new species on a phylogenetic tree by re-rooting the tree to the parent edge to predict the node [66]. We repeated this inference across 2,000 draws. Per draw, we randomly sampled 1 of the 100 trees. Then, we predicted the probability of each species to be assigned to each diet category by combining the predicted phylogenetic effects with the global intercept and slopes for maximum body size for each draw. Finally, we summarized all diet category probabilities per species by taking the mean and standard deviation across all 2,000 draws.

We quantified the total standard deviation (i.e., the square root of the quadratic sum of the standard deviations in each category) and the negentropy value, a measure of certainty calculated by subtracting 1 from the entropy value (i.e., uncertainty). Thus, the negentropy value lies between 0 and 1, and the higher the value, the higher the certainty for trophic guild assignment (i.e., if a given species has a high probability of assignment to a dietary category, the negentropy value will be high).

Finally, we conducted a cross validation to validate our extrapolation of trophic guilds to the global pool of fish species. Specifically, we repeated the extrapolation approach (as described above) 535 times, each time leaving out 1 species and predicting the trophic guild of that species. We then compared this prediction to the original assigned trophic guild and calculated the accuracy of each of the 8 trophic guild predictions.

### Prediction of trophic interactions with machine learning

To complement the assignment of discrete trophic guilds, we also modeled pairwise trophic interactions. In accordance with previous studies that infer trophic interactions by matching species traits or phylogenetic position [19,21,23,67,68], we predicted the probability of pairwise trophic interactions between the 535 reef fish species and the 38 prey categories in our dataset. Building on Laigle and colleagues [21], we developed a new machine learning approach to extract the reef fish phylogenetic tree from the Fish Tree of Life [61] and obtain phylogenetic eigenvector maps for each species, which were used as explanatory variables in our models [69]. We then predicted the probability of trophic interactions between fish species and prey categories based on phylogenetic position and maximum body size. Specifically, using the R package *h2o* [70], we employed an ensemble modeling approach based on 3 models calibrated with 10-fold cross validation: extreme gradient boosting [71], boosted regression trees [72], and random forest [73]. A cross-validated general linear model was used as a super-learner to create an optimal weighted average (i.e., an ensemble) of the predictions from the 3 models. The 3 models were implemented using 2,000 regression trees and default settings to reduce overfitting. Model performance was assessed using the area under the receiver operating characteristic curve (AUC) and true skills statistics (TSS) [74].

In addition to applying this analysis to our dataset, we also tested whether this technique could reliably predict pairwise trophic interactions for new species and locations. To this aim, we calibrated the models with only 5 locations, excluding the dataset from New Caledonia. We then used the New Caledonia dataset to assess model performance. As detailed above, after cross validation, we used our model to predict probabilities of pairwise trophic interactions between the 4,554 reef fish species and the 38 prey categories.

## Results

### Assessment of expert agreement

**Fig 1:**
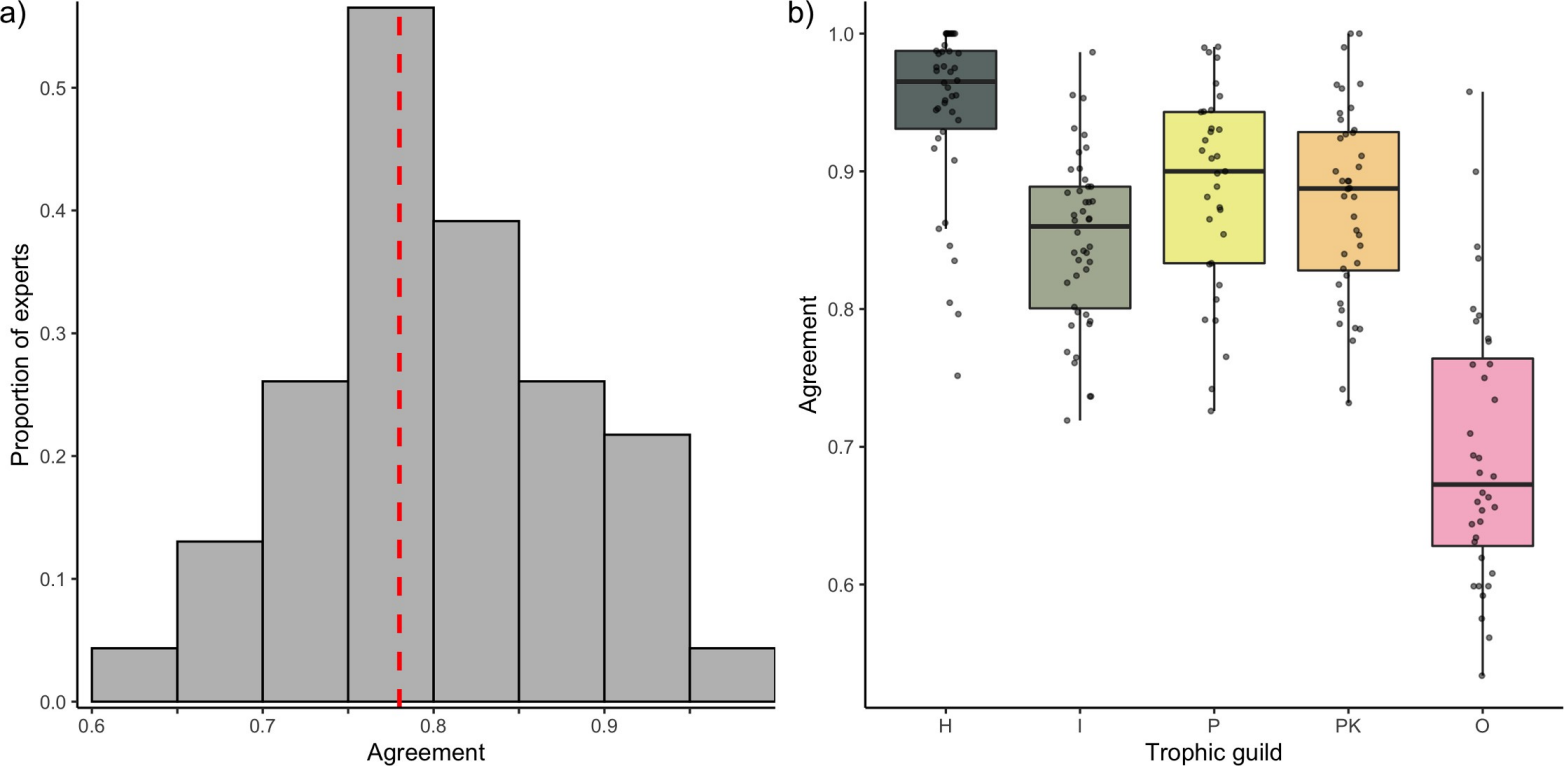
Expert agreement on trophic guild assignment. (**A**) The distribution of the agreement (i.e., proportion of species assigned to the same trophic category) across the 32 comparisons between pairs of experts. The red dotted line represents the median. (**B**) Agreement between pairs of experts by trophic category. The data underlying this figure may be found in https://github.com/valerianoparravicini/Trophic_Fish_2020. *H*, herbivores and detritivores; *I*, invertivores; *O*, omnivores; *P*, piscivores; *PK*, planktivores.

We evaluated expert agreement among 33 distinct and independent trophic guild classifications by comparing the classification schemes in pairs. Considering the broadness of the expert-assigned categories, we found low agreement. The median agreement between pairs, expressed as the proportion of species with matching trophic group assignments, was 78% (Fig 1). For approximately 50% of the pairwise comparisons, at least a quarter of the species were attributed to different trophic groups. In the most severe disagreement, the proportion of mismatched assignments reached 38%. In addition, expert agreement differed depending on the trophic group. Expert disagreement on the classification of “herbivores and detritivores” was low, with an average expert agreement of 95% and pairs of expert disagreement only reaching 20% (Fig 1B). In contrast, “omnivores” showed the highest mismatch, with experts agreeing on an average of only 70% of the species and peaks of disagreement between expert pairs reaching 47% (Fig 1B).

**Fig 2:**
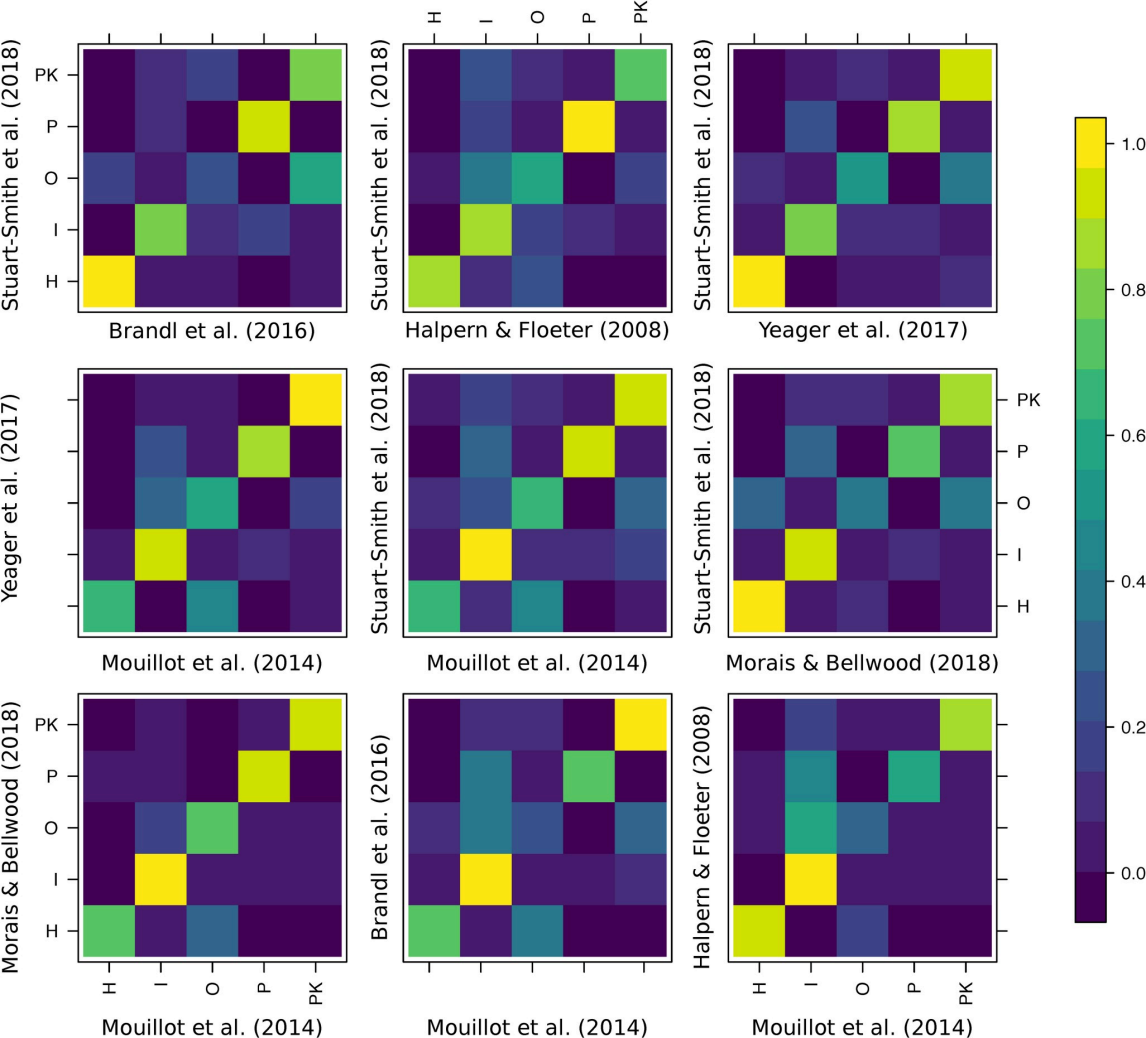
Confusion matrices of the agreement between pairs of experts that share at least 200 species in common and define all 5 trophic categories. Colors represent proportions of species in each trophic guild as classified by experts. The data underlying this figure may be found in https://github.com/valerianoparravicini/Trophic_Fish_2020. *H*, herbivores and detritivores; *I*, invertivores; *O*, omnivores; *P*, piscivores; *PK*, planktivores.

Expert agreement was variable and often homogeneously distributed around the mean for all the trophic categories. Therefore, the high agreement between a few combinations of experts did not necessarily exclude peaks of disagreement (Fig 1B). The analysis of individual confusion matrices between pairs of experts revealed high heterogeneity (Fig 2). For example, Morais and Bellwood were generally in agreement with Mouillot and colleagues [36] (across 89% of the 515 species in common), while Mouillot and colleagues [36] agreed with Stuart-Smith and colleagues [39] across only 68% of the 2,211 species in common.

Surprisingly, there was also a high heterogeneity in groups with high disagreement (i.e., multiple alternative assignments for species not assigned to the same trophic group). Species classified as “invertivores” according to 1 expert were considered “omnivores,” “piscivores,” or “planktivores” according to other classification schemes (Fig 2). Similarly, species considered “omnivores” by 1 expert were alternatively considered “invertivores,” “herbivores and detritivores,” or “planktivores” by another expert.

### Definition of trophic guilds with network analysis

**Fig 3:**
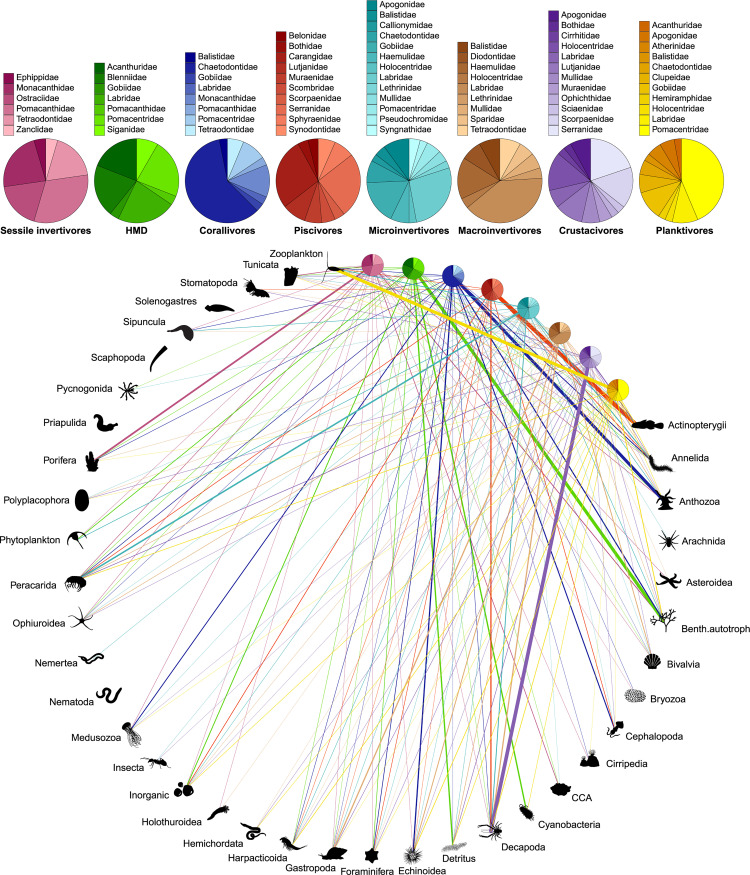
Bipartite network including 615 fish species (grouped into 8 trophic guilds) and their prey items (grouped into 38 categories; see S1 Table). The relative proportion of each prey category consumed by each trophic guild corresponds with the width of each interaction bar. The pie charts show the relative proportion of fish families within each trophic guild. The data underlying this figure may be found in https://github.com/valerianoparravicini/Trophic_Fish_2020. HMD, herbivores, microvores, and detritivores.

We defined trophic guilds by identifying modules (i.e., combinations of predators and prey) that maximize the weighted modularity of the global network [58]. Our analysis robustly identified 8 distinct modules that correspond to different trophic guilds (Fig 3). We identified these trophic guilds as

1. “Sessile invertivores”: species predominantly feeding on Asteroidea, Bryozoa, Cirripedia, Holothuroidea, Porifera, and Tunicata;
2. “Herbivores, microvores, and detritivores (HMD)”: species primarily feeding on autotrophs, detritus, inorganic material, foraminifera, and phytoplankton;
3. “Corallivores”: species predominantly feeding on Anthozoa and Medusozoa;
4. “Piscivores”: species primarily feeding on Actinopterygii and Cephalopoda;
5. “Microinvertivores”: species primarily feeding on Arachnida, Pycnogonida, small Crustacea (Peracarida), and worms (Annelida, Hemichordata, Nematoda, Nemertea, and Sipuncula);
6. “Macroinvertivores”: species primarily feeding on Mollusca (Bivalvia, Gastropoda, Polyplacophora, and Scaphopoda), Echinoidea, and Ophiuroidea;
7. “Crustacivores”: species primarily feeding on large Crustacea (Decapoda and Stomatopoda);
8. “Planktivores”: species mainly feeding on zooplankton, cyanobacteria and Harpacticoida.

### Phylogenetic conservatism of trophic guilds

To evaluate the significance of the phylogenetic statistic value (δ = 9.37), we applied a bootstrapping approach and quantified δ after randomly shuffling the trait values 100 times. The median δ of these null models was 0.000199 (95% confidence interval [0.000196, 0.000204]), indicating a strong phylogenetic signal associated with the 8 trophic guilds.

**Fig 4:**
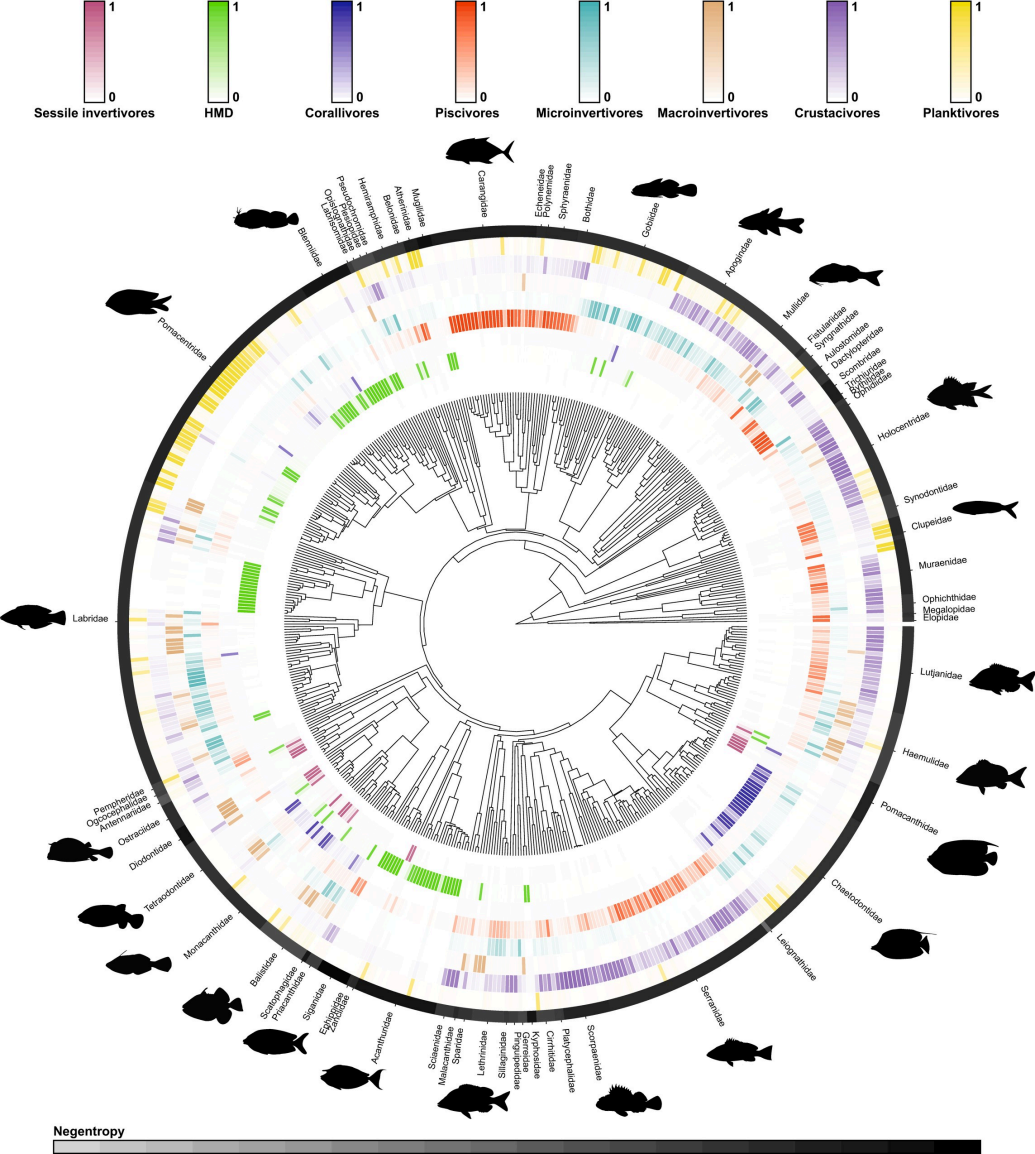
Phylogenetic tree of 535 reef fish species with fitted trophic guild assignments based on empirical dietary data. Trophic guild predictions were made with a Bayesian multinomial phylogenetic regression. The probability of trophic guild assignments for each species is visualized with color scales (depicted above the phylogenetic tree), with darker colors indicating a higher probability of assignment. In the outer black ring, each distinct segment represents a fish family (with silhouettes included for the most speciose families). Uncertainty of overarching trophic guild assignment for each fish family is visualized with negentropy values (i.e., reverse entropy); thus, darker shades indicate a higher degree of certainty of trophic guild assignment. Fish shapes are available at https://github.com/simonjbrandl/fishape/tree/master/shapes. The data underlying this figure may be found in https://github.com/valerianoparravicini/Trophic_Fish_2020.

Phylogeny and maximum body size were sufficient to correctly predict the trophic guild of 97% of the species in our dataset. For most families, there was strong phylogenetic conservatism, which resulted in the high confidence of these predictions (Fig 4). Within some families, however, closely related species displayed distinct dietary preferences, as showcased by high negentropy values for families such as Balistidae, Diodontidae, and Labridae.

Given its high predictive performance, we used our Bayesian phylogenetic model to extrapolate the probability of all reef fish species belonging to the 8 trophic guilds and assigned the trophic guild with the highest probability (S3 Table). Using leave-one-out cross validation, the final accuracy of this approach was 65%, which is comparable with other phylogenetically extrapolated traits applications, such as those involving microbial traits [75].

By inspecting the confusion matrix of the leave-one-out cross validation, we obtained more detailed information on the accuracy of the trophic guild predictions (S1 Fig). Most categories were well predicted with our extrapolation approach. In particular, the “sessile invertivores,” “HMD”, and “piscivores” trophic guilds were predicted with high accuracy (77%, 75%, and 73% correct predictions, respectively). The confusion matrix also provided information on incorrectly assigned categories. For example, when “piscivores” were incorrectly assigned, they were mostly classified as “crustacivores.” However, the network plot revealed that the fishes classified as “piscivores” also fed on crustaceans (mostly decapods), so this “incorrect assignment” was grounded in ecological reality and reflected uncertainty within the model. Additionally, the “microinvertivores” trophic guild had the highest proportion of inaccurate predictions (52% correct predictions). Here, species were often misclassified as “crustacivores” or “planktivores.”

### Prediction of trophic interactions with machine learning

Using machine learning, our model achieved high predictive performance in quantifying probabilities of pairwise trophic interactions (AUC = 0.99; TSS = 0.94). After calibration with 535 fish species and 3,479 trophic interactions, our model accurately identified 3,410 of these interactions, demonstrating an exceptionally low rate of false negative interactions. In addition, the model accurately predicted absent trophic interactions, with a false positive interaction rate of only 3.6%. When the model was calibrated with only 5 locations, excluding the data from New Caledonia, the model still performed well (AUC = 0.82; TSS = 0.52). The model correctly detected 82% of the trophic interactions in the New Caledonia dataset, with a false positive interaction rate of 27%. Based on the high predictive performance of the model, we used the full model with all 6 locations to predict the probability of pairwise trophic interactions on a continuous spectrum between the 4,554 reef fish species with available phylogenetic information and our 38 prey categories (S4 Table).

## Discussion

Functional ecology requires standardized and reproducible classification schemes to characterize species’ niches [76–78]. Rather than relying on expert opinion for the assignment of trophic groups, which often results in variable assignments, we demonstrate that the categorization of discrete trophic guilds and pairwise trophic interactions can be achieved with a quantitative, reproducible framework grounded in empirical data across biogeographic regions. We employed network analysis to partition 535 tropical coral reef fish species into 8 trophic guilds based on a synthesis of globally distributed, community-wide fish dietary analyses, and then we applied a Bayesian phylogenetic model that predicts trophic guilds based on phylogeny and body size, attaining a 5% misclassification error. Moreover, using a machine learning approach, we demonstrate that a continuous spectrum of trophic interactions can also be accurately predicted based on phylogeny and body size. Our framework represents the first implementation of a quantifiable classification scheme for coral reef fishes, which form some of the most diverse vertebrate communities worldwide.

Unlike traditional trophic guilds based on expert opinion [36,37,39,44–49], our trophic approaches are reproducible, provide uncertainty estimates, and can be updated and improved in the future with additional dietary information. In an effort to encourage new, accessible benchmarks to categorize fish trophic guilds, our classification of discrete trophic guilds and probabilities of pairwise trophic interactions are publicly available with this publication. Given the growing number of trait-based studies that assign trophic guilds to understand and monitor ecosystem functioning in our changing world, it is imperative that we establish comparable and reproducible trophic classification frameworks.

Our findings highlight the discordance of expert opinion in the assignment of trophic guilds and the necessity to develop quantifiable and reproducible classification schemes that are accessible to the wider scientific community (c.f. [79]). Despite broad similarities between the trophic guilds reported in the literature and the groups identified by our analysis, our classification scheme reveals a higher level of partitioning among invertebrate-feeding fishes as compared to previously proposed trophic guilds. In the past, invertebrate-feeding fishes were generally considered “sessile invertivores,” “mobile invertivores,” or “omnivores” (e.g., [37,38,48]), but we identify 5 distinct invertebrate-feeding groups: “corallivores,” “sessile invertivores,” “microinvertivores,” “macroinvertivores,” and “crustacivores.” Given the extreme numerical dominance of invertebrates in coral reef environments [80], the collapse of all invertebrate-feeders into 2 or 3 trophic groups was possibly an artefact of expert oversight, and the expansion of invertebrate-feeding trophic guilds to 5 groups stands to improve ecological resolution of fishes feeding on invertebrate prey.

In contrast to the high resolution achieved within invertebrate-feeding groups, our classification achieved limited resolution among the nominally herbivorous species, “HMD.” Across the literature, past classification schemes often separate macroalgal feeders, turf algae croppers, and detritivores (e.g., [36,37,40,41]). The lack of precision in our framework is rooted in the difficulty in distinguishing algae, microbes, and detritus within the alimentary tract of fishes, resulting in the pooling of these ingested items during the visual assessment of fish gut contents. Consequently, species classified as “HMD” may have fundamentally different foraging strategies, dietary preferences, and evolutionary histories [81], which can greatly impact their functional role on coral reefs (e.g., [82]). Thus, while our identified trophic guilds promise increased resolution for fishes that consume animal prey, our identified groupings may not adequately capture consumer–producer dynamics on coral reefs. Emerging techniques, such as gut content metabarcoding, may provide the additional resolution needed to further discriminate prey items in this group [83,84]. Alternatively, coupling diet categorization with other traits, such as feeding behavior, may help to pinpoint the variety of feeding modes that exist within the “HMD” trophic guild.

While our delineation of trophic guilds is applicable to functional studies that employ discrete categories, the continuous output of trophic interaction probabilities holds promise for a variety of other approaches, such as trophic network analyses. On coral reefs, previous studies have employed network analysis to examine human impacts on coral reef food webs [30,33]. However, these studies only incorporate local fish gut content data, which limits their spatial application. Larger-scale network analyses exist (e.g. [85]), but they are predominantly based on co-occurrence patterns and solely consider piscivores, thus neglecting a large portion of marine food webs, which are typically dominated by invertebrate-feeders. Therefore, our demonstrated ability to predict trophic interactions based on phylogeny and body size opens new avenues for marine food web research. Moreover, the high performance of the reduced model to predict pairwise trophic interactions in New Caledonia confirms the potential of our approach to predict probabilities of local trophic interactions for entire food webs.

Our findings add to recent evidence that evolutionary history (i.e., phylogenetic relatedness) is essential to evaluate the ecological traits of fishes (c.f. [86–88]). Recently, taxonomy and body size have been revealed as important predictors of fish diet composition and size structure [89,90], and in the highest resolution analyses of coral reef fish diet, taxonomic family was a better predictor of fish diet than broad trophic guilds [83]. Given the exceedingly low rate of misclassification error in our predictions, we posit that phylogeny is a critical variable that should be consistently considered in the assignment of trophic guilds for reef fishes. Across a plethora of organismal groups (e.g., birds [91], reptiles [92], fishes [93,94], insects [95], parasites [96], and plants [97]), phylogenetic niche conservatism has been alternately supported and dismissed. In our case, when examining fish trophic guilds using 38 prey categories, phylogenetic conservatism is readily apparent at a relatively coarse dietary resolution and may allow us to extrapolate trophic assignments to closely related consumer species and potentially extend this framework to fishes inhabiting other habitats. However, with increasing dietary resolution beyond what is detailed in the present study, phylogenetic signals may weaken [98] since even closely related species may exhibit dietary specialization [83,99]. In the future, with the availability of higher resolution of dietary information, phylogenetic niche conservatism can be easily examined within our framework.

With ongoing environmental and ecological change, a firm grasp on shifts in ecosystem functioning will depend on the reliable assignment of organismal traits [15] and the comparability of trait-based approaches across space, time, and independent studies [77]. Especially in complex, hyperdiverse environments such as coral reefs, it is imperative to standardize how we measure and report these traits to prevent idiosyncratic results based on subjective trait assignments [27,100]. Trophic guilds are among the most commonly applied trait to assess ecosystem functioning because they directly relate to energy and nutrient fluxes across trophic levels. Thus, our standardized framework represents a major step forward for coral reef functional ecology, while heeding the call for openly accessible, reproducible trait databases [31,78,101]. As trait-based ecology continues to be used to examine disturbances and implement management strategies, our cohesive and accessible framework can provide key insights into the trajectory of coral reef communities.

Further, our results can serve as the foundation for an online platform that permits researchers to collate, update, and utilize trait-based data on coral reef fishes. Similar to current initiatives across the entire tree of life [78], the creation of an online, user-maintained dietary database will facilitate collaboration and traceability in trait-based reef fish research. One challenge will lie in merging visual fish gut content analysis databases with molecular data, such as gut content DNA metabarcoding (e.g., [83]), and biochemical data, such as stable isotope analysis (e.g., [102]), and short-chain fatty acid profiles (e.g., [103]), which indicate nutritional assimilation rather than the simple ingestion of prey items [81]. Despite this challenge, accessibility to a large breadth of reef fish dietary information would improve our framework. Our proposed trophic guilds and probabilities of trophic interactions are model predictions, so they are only as reliable as the underlying dietary data. In addition, these predictions may suffer from extrapolation biases; for example, if limited dietary information exists across species within a taxonomic group, extrapolations to closely related species are more likely to be assigned erroneous trophic guilds. Consequently, an ongoing, extensive compilation of dietary traits across coral reef fishes will continuously improve our predicted trophic guild assignments and pairwise trophic interactions.

Finally, our proposed framework is not limited to coral reef fishes; indeed, trophic guild assignments can be quantifiable, reproducible, and transparent, with the inclusion of uncertainty metrics, across many organismal groups. However, the standardization of trophic guilds is sorely lacking across the ecological literature [79], especially based on quantitative data (e.g., [104]). We posit that a similar approach can be readily applied across a multitude of organisms and environments. In fact, given the paucity of dietary information available for coral reef fishes in comparison to other organisms, particularly birds and mammals, building rigorous, global trophic classification schemes for many other organisms should be readily achievable within our framework. With a quantitative, transparent trophic classification scheme that can be augmented over time and is applicable across ecological systems, our framework represents a significant advancement for trait-based ecology and a viable approach to monitor ecosystem dynamics into the future [78].

## Supporting information

S1 Fig Confusion matrix showcasing the accuracy of the 8 trophic guild predictions from the leave-one-out cross validation based on the extrapolation of the Bayesian phylogenetic model. Trophic guilds include (1) “sessile invertivores,” (2) “herbivores, microvores, and detritivores,” (3) “corallivores,” (4) “piscivores,” (5) “microinvertivores,” (6) macroinvertivores, (7) “crustacivores,” and (8) “planktivores.” The data underlying this figure may be found in https://github.com/valerianoparravicini/Trophic_Fish_2020.
(PNG)

S1 Table Prey categories used to define the trophic guilds of coral reef fishes. (CSV)

S2 Table Summary of the 33 papers used to evaluate expert agreement on reef fish trophic guilds. The column named “Fishes” refers to the number of fish species included in that study.
(CSV)

S3 Table Global extrapolation to infer the probability of each of the 4554 reef fish species to belong to the 8 trophic guilds. The mean and the standard deviation (e.g., p1-8_m, and p1-8_sd) of the posterior probabilities are reported alongside with the mean and standard deviation of the negentropy.
(CSV)

S4 Table Probability of trophic interaction between the 4554 reef fish species and the 38 prey categories according to the extrapolation performed by the machine learning approach. (CSV)

## Abbreviations

AUC: area under the receiver operating characteristic curve
HMD: herbivores, microvores, and detritivores
TSS: true skills statistics
