# Supplementary figures and images for: Delineating reef fish trophic guilds with global gut content data synthesis and phylogeny
Source: PLoS Biol. 2020 Dec 28;18(12):e3000702. doi: 10.1371/journal.pbio.3000702 (PMC7793298; doi:10.1371/journal.pbio.3000702)

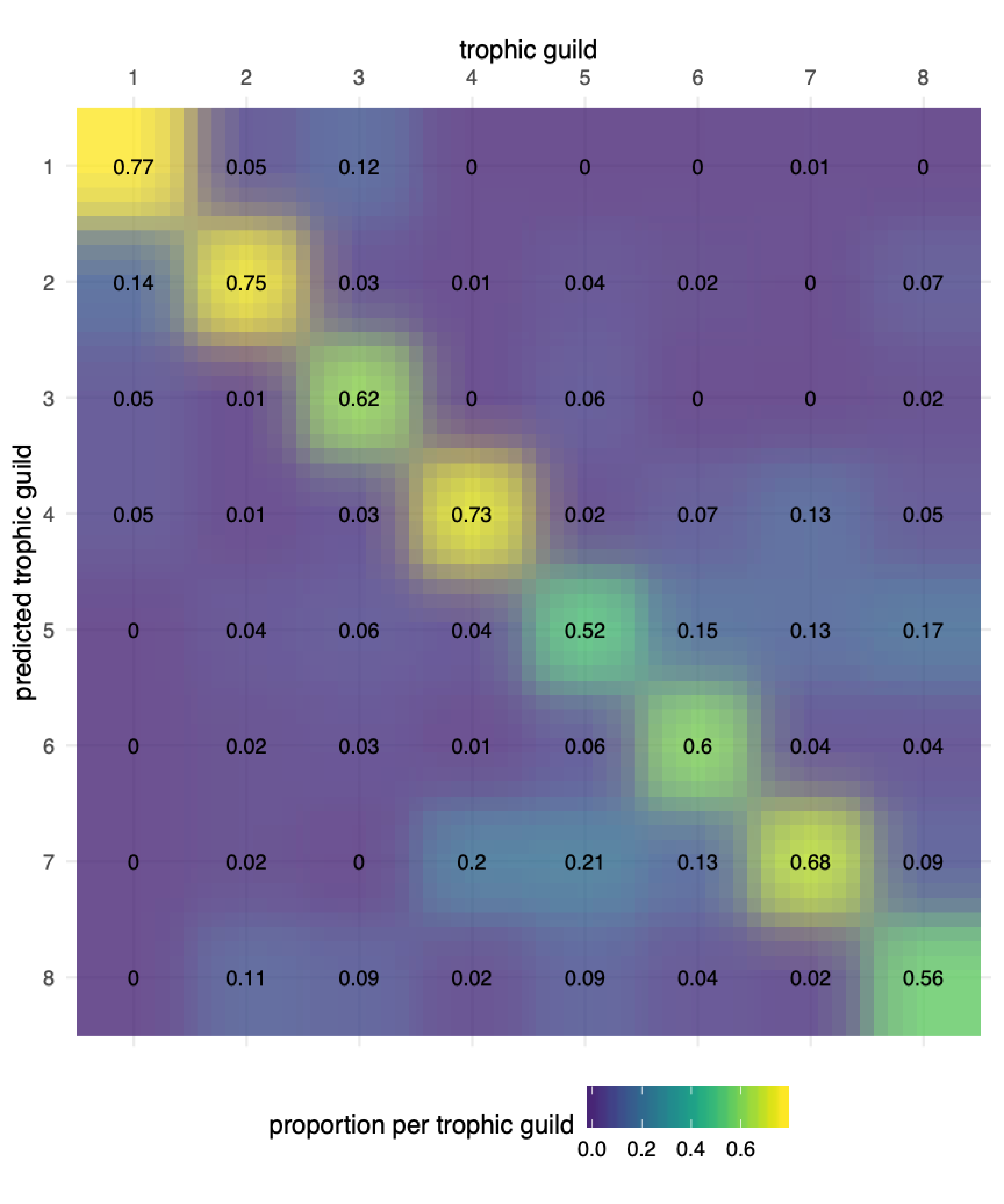

Supplement: S1 Fig — Trophic guilds include (1) “sessile invertivores,” (2) “herbivores, microvores, and detritivores,” (3) “corallivores,” (4) “piscivores,” (5) “microinvertivores,” (6) macroinvertivores, (7) “crustacivores,” and (8) “planktivores.” The data underlying this figure may be found in https://github.com/valerianoparravicini/Trophic_Fish_2020. (PNG) [file pbio.3000702.s001.png]
